# Near-Field Direct Writing Based on Piezoelectric Micromotion for the Programmable Manufacturing of Serpentine Structures

**DOI:** 10.3390/mi15121478

**Published:** 2024-12-07

**Authors:** Xun Chen, Xuanzhi Zhang, Jianfeng Sun, Rongguang Zhang, Xuanyang Liang, Jiecai Long, Jingsong Yao, Xin Chen, Han Wang, Yu Zhang, Jiewu Leng, Renquan Lu

**Affiliations:** 1 State Key Laboratory of Precision Electronic Manufacturing Technology and Equipment, Guangdong University of Technology, Guangzhou 510006, China; xunchen@gdut.edu.cn (X.C.); xuanzhizhang@foxmail.com (X.Z.); scien2023@163.com (J.S.); zrg941230@163.com (R.Z.); yjsong163yx@163.com (J.Y.); chenx@gdut.edu.cn (X.C.); wanghangood@gdut.edu.cn (H.W.); zhangyu@gdut.edu.cn (Y.Z.); jwleng@gdut.edu.cn (J.L.); 2 School of Electromechnical Engineering, Guangdong University of Technology, Guangzhou 510006, China; a1744631037@163.com; 3 Guangdong Provincial Key Laboratory of Intelligent Decision and Cooperative Control, School of Automation, Guangdong University of Technology, Guangzhou 510006, China; rqlu@gdut.edu.cn

**Keywords:** near-field direct writing (NFDW), nanopositioning, piezoelectric ceramics (PZT), fiber deposition, serpentine microstructure

## Abstract

Serpentine microstructures offer excellent physical properties, making them highly promising in applications in stretchable electronics and tissue engineering. However, existing fabrication methods, such as electrospinning and lithography, face significant challenges in producing microscale serpentine structures that are cost-effective, efficient, and controllable. These methods often struggle with achieving precise control over fiber morphology and scalability. In this study, we developed a near-field direct writing (NFDW) technique incorporating piezoelectric micromotion to enable the precise fabrication of serpentine micro-/nanofibers by incorporating micromotion control with macroscopic movement. Modifying the fiber structure allowed for adjustments to the mechanical properties, including tunable extensibility and distinct characteristics. Through the control of the frequency and amplitude of the piezoelectric signal, the printing errors were reduced to below 9.48% in the cycle length direction and 6.33% in the peak height direction. A predictive model for the geometrical extensibility of serpentine structures was derived from Legendre’s incomplete elliptic integral of the second kind and incorporated an error correction factor, which significantly reduced the calculation errors in predicting geometric elongation, by 95.85%. The relationship between microstructure bending and biomimetic non-linear mechanical behavior was explored through tensile testing. By controlling the input electrical signals, highly ordered serpentine microstructures were successfully fabricated, demonstrating potential for use in biomimetic mechanical scaffolds.

## 1. Introduction

Microscale wave-like/serpentine structures have gained prominence as a research focus due to their unique properties and diverse applications in fields such as stretchable electronic devices [1], tissue engineering [2], actuators [3], and human–machine interfaces [4]. Serpentine constructions are particularly well suited to these domains due to their distinctive capacity to withstand substantial deformations without fracturing. The integration of serpentine structures into flexible devices allows for the maintenance of functionality even under conditions of bending or stretching, which is a highly desirable quality for stretchable electronic devices [5]. In the field of tissue engineering, serpentine structures demonstrate remarkable potential for modeling the mechanical behavior of biological tissues, including tendons and ligaments [6]. These tissues are highly malleable and exhibit non-linear mechanical responses, a property that is particularly advantageous when designing scaffolds to support cell growth and tissue regeneration. The inherent flexibility and fatigue resistance of the serpentine design permit it to withstand repeated cycles of stretching and compression, thereby making it an optimal choice for applications where mechanical durability is of paramount importance. For example, a bridge structure with a serpentine design can achieve ~100% ductility [7]. Xu et al. [8] have integrated the serpentine structure into a high-charge flexible battery that can be recharged wirelessly, resulting in a 300% elongation.

Among various fabrication techniques, electrospinning stands out for its efficiency and environmentally friendly production of micro- and nanofibers [9]. Advanced methods such as electron beam lithography [10], focused ion beam (FIB) [11], and dip-pen nanolithography (DPN) [12] can also be used to fabricate these structures; however, these techniques typically require specialized equipment and complex processes, which lead to high costs and lengthy production times. In contrast, near-field electrospinning (NFES) or near-field direct writing (NFDW) [13] enables 3D printing-like precision by reducing the inter-electrode distance. This modification minimizes jet oscillations and suppresses chaotic fiber trajectories, significantly enhancing deposition control. This control enables the direct writing of smooth microfibers with precise trajectories and enhanced mechanical properties, facilitating the design of scaffolds with specific geometries and offering promising applications in tissue engineering [14,15,16]. Previous studies have indicated that the structural parameters and mechanical properties of electrospinning fibers can influence cell behavior [17,18]. For instance, Xie et al. [19] explored the feasibility of inducing cell growth using fibrous scaffolds with different fiber diameters and pore sizes, revealing higher cell growth rates in scaffolds with smaller pore sizes, particularly when the cell diameter matched the fiber diameter. This aspect was further elucidated by Zhang et al. [20], who showed that cell orientation depends on the matching degree between the pattern feature size and the cell’s minor axis. Typically, these micron-level dimensions align with the conventional technical indicators of NFDW. Xiong et al. [21] investigated the influence of fiber ”waviness” on cell orientation, revealing that increasing the fiber orientation angle (i.e., the degree of waviness) enhances cell alignment. However, achieving high-precision controllable fiber microstructures through NFDW remains more challenging.

The researchers aimed to enhance near-field direct writing (NFDW) technology to achieve controllable waveform microstructures capable of satisfying demand. Previous studies had predominantly explored three main strategies for fabricating serpentine microstructures: (i) the axial-compression bending approach based on jet buckling instability [22]; (ii) physical field-assisted methods using external electric or magnetic fields, and (iii) the pre-stretched substrate method. For instance, Duan et al. [23] employed the first approach to fabricate various winding structures, including spirals, alternating rings, and meandering patterns, using geometric models to explain the transformation rules among different modes. However, these structures were produced passively, with the fiber deposition shape being largely dictated by the process parameters and fluid characteristics, thus limiting the precision in adjusting the structural parameters. The second strategy has attracted more attention among researchers for printing applications. For example, Fang et al. [24] leveraged the intrinsic charge of the jet by applying two lateral electric fields to control jet deflection during its flight, enabling the adjustment of the frequency, amplitude, and wavelength of the resulting pattern through the precise control of the auxiliary electric field’s size and frequency. On this foundation, Zhu et al. [25] derived a formula to calculate the generation frequency of wavy fibers and highlighted the potential of this technique for grating encoding applications. Furthermore, You et al. [26] expanded this approach by incorporating four lateral auxiliary electrodes around the nozzle to achieve more precise control of jet deflection. A theoretical model was developed, building on the work of Reneker et al. [27], to analyze the behavior of the jet trajectory under uneven electric field distributions in near-field electrospinning. This model provided guidance for the practical application of the technique, allowing for the precise calculation of deposition trajectories. Nevertheless, the approach of manipulating the electric field has significant limitations, particularly because it can only control the deflection of a single jet. As the demand for higher application throughput grows, we are seeing a shift toward mass production in near-field direct writing, with one straightforward solution being an increase in the number of nozzles. Wang et al. [28] proposed a multi-nozzle NFDW technique to systematically investigate the deposition characteristics of multi-nozzle direct writing, achieving highly consistent parallel fibers. However, when the number of jets increases, the resulting jet competition complicates the spatial electric field, making the use of the physical field manipulation method challenging in the high-throughput, large-scale manufacturing of microstructures. Duan et al. [29] utilized the third strategy to achieve the mass production of highly extended meandering fiber structures through a buckling mismatch between the electrospinning fiber and the substrate. However, this manufacturing approach lacks intuitiveness, as constructing an accurate mathematical relationship between the process parameters and the structural parameters is challenging. On the other hand, the aforementioned methods ensure the controllability of the printing process through indirect means. However, due to the limitations of the platform’s own driving components, these methods have a limited gain effect on most low-precision platforms available on the market. To date, there have been no reports that demonstrate the ability to achieve fiber-controlled direct writing with both large travel and microstructural precision.

The macro–micro hybrid motion approach combines macro-scale actuators with micro-precision actuators, effectively resolving the conflict among large travel, high acceleration, and high precision, and thereby demonstrating exceptional performance [30]. PZT-based micromotion platforms are common micro-precision actuators with widespread applications in microtechnologies [31,32,33]. For example, Xu et al. [34] designed a novel asymmetric flexible micro-gripper based on piezoelectric ceramics, which could easily achieve submicron-level gripping actions. Lu et al. [35] proposed a three-degree-of-freedom focusing system based on a folding mirror, utilizing piezoelectric ceramics as the actuator, which not only achieved precise focusing, but also significantly reduced the size and weight of the focusing mechanism. These platforms sacrifice large travel in order to achieve micron and nanoscale positioning and manipulation, introducing a new concept for controlling the micron–nano-scale formation of electrospinning fiber patterns.

In this paper, we propose and validate a novel, versatile manufacturing strategy for programmable bending microstructures. By integrating a high-precision PZT-driven nanopositioning platform into a near-field direct writing (NFDW) system, this approach leverages a macro–micro hybrid control concept. Through the combination of PZT-driven micromovements and the macroscopic motion of the NFDW system, our approach enables the fabrication of large-scale, arbitrary wave-like and serpentine microstructure patterns. Due to the extensive research on micromotion platform design [36,37,38,39,40], this strategy is easily adaptable to various direct-writing platforms, demonstrating high versatility. Initially, the consistency and trajectory controllability of the printed fibers were examined to confirm the feasibility of the proposed strategy. To guide the programmable fabrication of serpentine structures, a theoretical model was developed to describe the relationship between the jet trajectory and structure extensibility. The impact of process parameters on the geometry of the deposited patterns was analyzed. The capability of this method was further validated through the fabrication of various microscale wave-like and serpentine structures. Finally, the relationship between the degree of bending in serpentine structures and their non-linear mechanical behavior was explored.

## 2. Materials and Methods

### 2.1. Principle of Serpentine Track Generation

In this study, we present an innovative NFDW fabrication method that incorporates controlled reciprocating micromotions to produce serpentine microstructures. This approach relies on electric field-driven jet motion and the precise control of the micromotion platform, enabling accurate microstructure fabrication.

**Figure 1:**
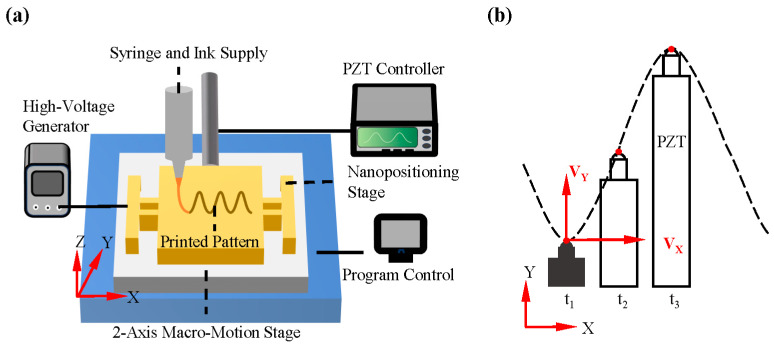
(**a**) Schematic diagram of the macro–micro-composite-driven motion platform; (**b**) the principles of serpentine trajectory modeling.

The fundamental configuration of the device is illustrated in Figure 1a, which depicts the integration of essential components, including a high-voltage generator, a spinning nozzle, and a motion-programmable receiving platform. Additionally, a micromotion platform based on a flexible mechanism has been designed and integrated, which represents a significant advancement over conventional NFDW systems. The majority of direct-writing systems currently in use directly employ commercial printing platforms. While these platforms are capable of achieving significant stroke displacement, their printing accuracy is typically limited to 100 μm, due to cost considerations [41]. This is insufficient for the scaling requirements for microstructure fabrication. In contrast, the resolution of PZT actuators can reach the nanometer level, offering a potential avenue for improving the printing accuracy by an order of magnitude. The longitudinal motion of the jet is regulated by a vertical electric field applied to the nozzle, while the transverse trajectory is controlled by the micromotion platform and the 2D macro-motion platform, respectively. This control strategy combines the substantial stroke displacement of the macro-motion platform with the precision micromotion of the PZT-driven platform, significantly simplifying programming control. Figure 1b illustrates the principle of trajectory synthesis, where the red dot and dotted line represent the moving point of the PZT and the moving track composed of the moving point, respectively. During the transverse uniform displacement of the macro-actuated platform, the PZT-driven micro-actuated platform produces electro-induced pulse micro-displacement in the orthogonal direction, and its mathematical expression is shown in Equation (1).

(1) $$\left\{\begin{array}{c} X=vt\\ Y=A\sin\left(\omega t\right) \end{array}\right.$$

*v* is the motion velocity of the macro-motion platform, *A* and *ω* are the amplitude and frequency of the pulse motion, respectively, and *t* is the motion time. This micro-displacement, combined with the X-directional displacement of the macro-actuated stage, creates a meander-like trajectory. In some cases, even if the micro-displacement applied in the orthogonal direction is not a sinusoidal waveform but a linear iso-acceleration motion, the displacement can always be described as a sine wave, due to the existence of the “hysteresis effect”, so Equation (1) always applies. The “hysteresis effect” will be discussed in more detail later.

### 2.2. Theoretical Modeling

In order to create a universal method, this paper presents a model for predicting the ductility of serpentine microstructures based on Legendre’s elliptic integral of the second kind. By involving the control variables *A* and *ω*, i.e., the output amplitude and frequency of the PZT actuator, the programmable fabrication of the microstructures becomes possible. The accuracy of the model was verified in subsequent experiments. At the same time, a correction coefficient was introduced, to take into account possible errors in the printing process. This coefficient is obtained by calculating the error value arising between the model and the actual structure and is used to correct the theoretical model derived as follows.

First, the serpentine structure is abstracted into a plane smooth curve arc *L*, which can be determined by the parametric Equation (2).

(2) $$\left\{\begin{array}{c} X=x\left(l\right)=l\\ Y=A_{out}\sin\left(\frac{2\pi w_{in}}{V_{cl}}x\left(l\right)\right) \end{array}\right.$$

Here, $A_{out}=KA_{in},\;f=\frac{2\pi w_{in}}{V_{cl}}$, and l is the independent variable of the parametric equation, where $A_{in}$ is the input amplitude of the PZT actuator, affecting the peak height of the serpentine structure; $\omega_{in}$ is the input frequency, affecting the cycle length of the serpentine structure; and $V_{cl}$ is the speed of the macro-actuated platform. K is the displacement amplification of the nanopositioning stage. Due to the characteristics of the PZT actuator, $A_{in}>0$ and f>0.

To derive the length of the curve arc *L*, we can use the product formula $S=\int_{\alpha }^{\beta }\sqrt{{X^{\prime }}^{2}\left(t\right)+{Y^{\prime }}^{2}\left(t\right)}dt$ as follows:

(3) $$L=\int_{X_{1}}^{X_{2}}\sqrt{1+{A_{out}}^{2}f^{2}\cos^{2}fl}dl$$

This is a definite integral, and it is clear that the curve is continuous in the range $L:\left[x_{1},x_{2}\right]$, provided that $x_{2}\geq x_{1}\geq 0$ and $S=x_{2}-x_{1}$, where the exact values of $x_{1}$ and $x_{2}$ are specified artificially by the experimenter and are usually taken to be the coordinates of the points at the ends of the microstructure.

According to the trigonometric property $\sin^{2}x+\cos^{2}x=1$, the above equation can be simplified as follows:

(4) $$L=\int_{0}^{x_{2}}\sqrt{\left(1+{A_{out}}^{2}f^{2}\right)-{A_{out}}^{2}f^{2}\sin^{2}fl}dl\;-\int_{0}^{x_{1}}\sqrt{\left(1+{A_{out}}^{2}f^{2}\right)-{A_{out}}^{2}f^{2}\sin^{2}fl}dl$$

We define u=fl and substitute this into Equation (4) to derive the following:

(5) $$L=\frac{\sqrt{1+{A_{out}}^{2}f^{2}}}{f}[\int_{0}^{{fx}_{2}}\sqrt{1-\frac{{A_{\mathrm{out}}}^{2}f^{2}}{1+{A_{\mathrm{out}}}^{2}f^{2}}\sin^{2}u}du\;-\int_{0}^{fx_{1}}\sqrt{1-\frac{{A_{\mathrm{out}}}^{2}f^{2}}{1+{A_{\mathrm{out}}}^{2}f^{2}}\sin^{2}u}du]$$

Obviously, $\frac{{A_{out}}^{2}f^{2}}{1+{A_{out}}^{2}f^{2}}$ in Equation (5) must satisfy the condition of being less than 1. Therefore, it is obtained according to Legendre’s incomplete elliptic integral of the second kind [42]:

(6) $$E\left(k,\varphi \right)=\int_{0}^{\varphi }\sqrt{1-k^{2}{sin}^{2}\theta }\;d\theta$$

In particular, the *k* of the above equation should be satisfied as $k^{2}<1$, from which we can obtain the following:

(7) $$L=\frac{\sqrt{1+{A_{out}}^{2}f^{2}}}{f}\left[E\left(\frac{{A_{out}}^{2}f^{2}}{1+{A_{out}}^{2}f^{2}},{fx}_{2}\right)-E\left(\frac{{A_{out}}^{2}f^{2}}{1+{A_{out}}^{2}f^{2}},{fx}_{1}\right)\right]$$

In particular, it is customary to set the starting point $X_{1}$ to 0 when performing curve length calculations within a certain interval, and so the above equation can be further simplified as follows:

(8) $$\int_{0}^{x_{2}}\sqrt{1+{A_{out}}^{2}f^{2}\cos^{2}fl}dl=\frac{\sqrt{1+{A_{out}}^{2}f^{2}}}{f}E\left(\frac{{A_{out}}^{2}f^{2}}{1+{A_{out}}^{2}f^{2}},fS\right)$$

Here, we define $\varepsilon =A_{out}f$, and the final formula is given in the following equation:

(9) $$R_{L}=\frac{\frac{\sqrt{1+\varepsilon^{2}}}{f}E\left(\frac{\varepsilon^{2}}{1+\varepsilon^{2}},fS\right)}{S}$$

Meanwhile, to further improve the accuracy of the model, the correction coefficients $\mu_{f}$ and $\mu_{p}$ are set to correspond to the period output error and amplitude output error, respectively. The reason for setting the correction coefficients will be discussed in the Results section. After this, the corrected output amplitude $\bar{A_{out}}$ and frequency $\bar{f}$ are as shown in the following equation:

(10) $$\bar{A_{out}}=KA_{in}\left(1-\mu_{p}\right)$$

(11) $$\bar{f}=\frac{2\pi w_{in}}{V_{cl}\left(1-\mu_{f}\right)}$$

(12) $$\bar{S}=S\left(1-\mu_{f}\right)$$

(13) $$\bar{\varepsilon }=\bar{A_{out}}\times \bar{f}$$

Then, the final ductility formula is obtained as follows:

(14) $$R_{L}=\frac{\frac{\sqrt{1+{\bar{\varepsilon }}^{2}}}{\bar{f}}E\left(\frac{\varepsilon^{2}}{1+\varepsilon^{2}},\bar{f}\bar{S}\right)}{\bar{S}}$$

### 2.3. System Setup

**Figure 2:**
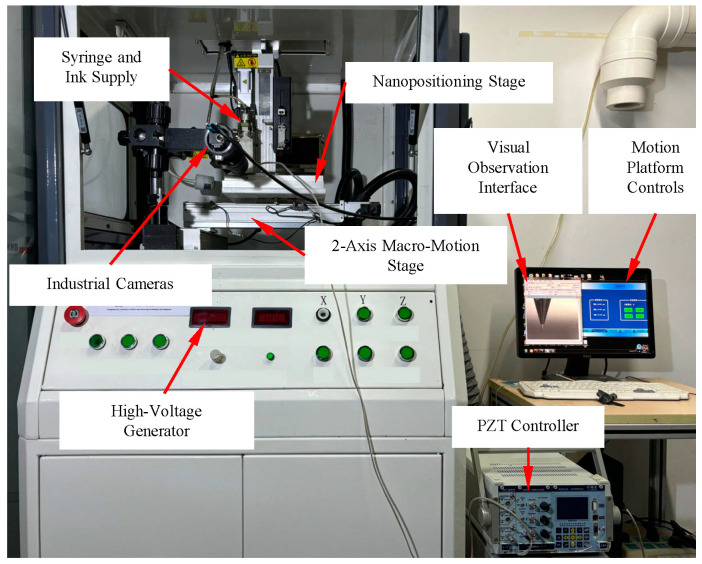
Self-built NFDW electrospinning experimental equipment.

A custom-built electrospinning system was developed to achieve precise control over the fiber trajectories, as shown in Figure 2. The performance parameters of the micromotion platform are detailed in Table 1. The PZT actuator (PSt150/7/100VS12, Harbin Core Tomorrow Technology, Inc., Harbin, China) used in the experiments has a nominal stroke of 95 μm and a displacement resolution of 7 nm, with a theoretical micro-displacement exceeding 290 μm. Operated in a closed-loop system with a self-contained controller, it offers various drive modes (sinusoidal, triangular, etc.), selectable through the control interface. The polymer melt was controlled via an electrical heating system around a syringe, with the precise polymer supply regulated by a pneumatic pump. ITO conductive glass, chosen for its low surface resistance and smooth flatness, serves as the substrate for fiber collection. A constant high voltage was applied to the needle by a high-voltage supplier (DW-N303-100ACC2, Tianjin Dongwen High-Voltage Power Supply, Inc., Tianjin, China). The entire printing process was monitored and recorded using a CMOS camera (DMK33UX265, The Imaging Source, Inc., Bremen, Germany).

### 2.4. Feasibility Verification and Control Variable Analysis

During the manufacturing process, the input amplitude of the piezoelectric ceramic actuator significantly affects the jet oscillation amplitude. The input electrical-signal frequency directly correlates with the jet oscillation frequency, while the signal waveform determines the motion characteristics of the piezoelectric ceramics. To verify the effectiveness and feasibility, a comprehensive control variable analysis was conducted to examine the influence of each variable. Three experimental sets were designed to study the effects on sedimentary pattern geometry, focusing on the amplitude, period, and ductility of the serpentine structures.

In the first group (Group 1), only the input signal amplitude was varied, among 40, 50, 60, and 70 μm. The selection of this amplitude range (40–70 μm) was based on application-specific requirements. Previous studies have shown that different pore sizes have varying effects on different cell types [43]. For example, pore sizes of 100–400 μm are commonly used for bone regeneration, while 45–150 μm are more suitable for liver tissue regeneration. Notably, large pores with diameters greater than 100 μm are essential in cell proliferation and the formation of related blood vessels [44]. Considering these factors, along with the stroke of the PZT actuators, the 40–70 μm range was selected, resulting in a platform displacement between 120 and 210 μm, which aligns with potential application needs. In the second group (Group 2), only the input signal frequency was changed, with the values set at 1, 2, 4, and 6 Hz. Changing the signal frequency allows for the adjustment of the structural period length, which is also dependent on the varying collection speeds. Due to the relatively high platform movement speed used in the experiments, even small changes in frequency can have a significant impact on the period length. As a result, lower frequencies (1–6 Hz) were selected, and different input frequencies with varying intervals were applied to demonstrate precise control over the structural parameters. In the third group (Group 3), only the signal waveform was modified, utilizing sine, square (with varying peak heights), sawtooth, and triangle waves for structure printing. For the first two groups, we used sinusoidal signals as examples.

### 2.5. Material Selection and Pattern Measurement

In the experiment, thermoplastic PCL pellets (CapaTM 6800, Perstorp, Shanghai, China, melting point 65 °C), widely used in electrospinning, were selected and processed using a Musashi needle (size 25, inner diameter 0.25 mm). PCL was chosen due to its excellent biodegradability and biocompatibility, which make it a widely used biodegradable material whose safety has been proven in numerous studies [45,46]. Additionally, its low melting point, good thermal stability, and excellent electrospinning processability make it one of the few thermoplastic materials suitable for NFDW.

Micropatterns were observed with a scanning electron microscope (TM3030, Hitachi, Ltd., Tokyo, Japan) operating at an accelerating voltage of 15 kV. SEM images were used to assess the structural parameters of fiber micropatterns, focusing on their amplitude, period, and ductility. ImagePro Plus 6.0 soft imaging system was employed to measure the structural parameters from at least 10 different sections of each sample, reducing the error by averaging the values.

### 2.6. Mechanical Testing of Serpentine Structure Fibers

To measure the mechanical properties of the fibers, five equidistant, parallel fiber patterns were printed on ITO conductive glass. The serpentine structures were then removed from the ITO glass by soaking in ethanol, followed by drying in a 45 °C vacuum oven for 30 min before mechanical testing.

Notably, fibers with different serpentine structures exhibit distinct mechanical properties. To validate this observation, serpentine structures with input amplitudes of 40, 50, 60, and 70 μm; frequencies of 1, 2, 4, and 6 Hz; and lengths of 2 cm were printed for tensile testing. Uniaxial tension was applied simultaneously to five curved structures using a tensile testing machine (CMT2000, SUST, Zhuhai, China) at a displacement rate of 10 mm/min until failure. Unlike conventional mechanical tests, this experiment was focused on determining the variation in the length of the stress toe region by analyzing the stress–displacement curve. This region is defined as the segment length from the displacement zero point to the starting point of elastic modulus calculation.

## 3. Results and Discussion

### 3.1. Feasibility Verification

**Figure 3:**
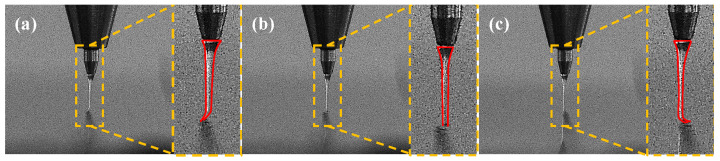
(**a**) Jet deflection when the PZT is retracted; (**b**) jet in a neutral position when the PZT is in the initial position; (**c**) jet deflection when the PZT is extended.

To generate specific waveforms, electrical signals were applied to the PZT actuator. Modulating the input signals caused corresponding changes in the PZT actuator’s behavior. This precise control governs the degree of meandering and the shape of the pattern deposition trajectory. As shown in Figure 3, applying electrical signals such as sine, square, or triangular waves enabled the observation of lateral jet vibrations driven by PZT micro-actuation, where the motion profile of the jet is depicted with a red frame.

To ensure stability and precision during printing, specific protocols were followed to determine the optimal parameters, with a focus on mitigating axial-compression bending and jet deflection. Under established equipment and environmental conditions, the essential parameters for optimal spinning included the electrode spacing, voltage, air pressure, and platform collection velocity.

**Figure 4:**
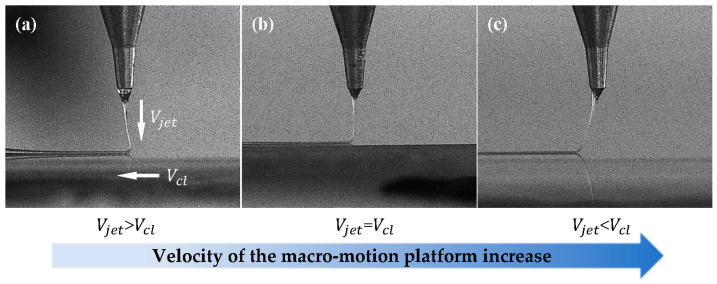
The velocity-matching mechanism between the jet and collector: (**a**) the jet state when $V_{jet}\;$>$\;V_{cl}$; (**b**) the jet state when $V_{jet}\;$=${\;V}_{cl}$; (**c**) the jet state when $V_{jet\;}$<${\;V}_{cl}$.

In the NFDW process, the voltage is crucial. Increasing the voltage intensifies the jet’s erratic oscillation, posing challenges in terms of direct control. Therefore, a narrower electrode spacing (typically 2 mm or less) is preferred, to minimize the voltage and prevent instability. Lower pressures enhance morphology control but lead to the risk of supply interruption, while higher pressures may cause melt suspension. The stability of the volume of the Taylor cone in the spinning process can be used as a criterion in regulating the air pressure. Subsequently, harmonizing the voltage with the platform collection velocity emerges as the linchpin for stable and controllable fiber trajectories. Figure 4 illustrates the alignment mechanism and shows that, whenever the platform velocity is less than that of the jet (i.e., $V_{jet}\;$>$\;V_{cl}$), then axial compression and bending cause “telephone line” or irregular wave-type fiber deposition. Conversely, when it matches the jet speed (i.e., $V_{jet}\;$=${\;V}_{cl}$), minimal jet deflection ensues, signaling the pivotal phase for optimal direct-writing controllability. If the platform speed surpasses the jet speed (i.e., $V_{jet\;}$<${\;V}_{cl}$), then a “hysteresis effect” induces excessive fiber stretching and straightening. Higher platform acceleration is recommended to mitigate non-linearity in platform motion and jet instability. Based on the aforementioned discussion, the spinning parameters for this experiment are shown in Table 2. Subsequent experiments will incorporate these data under standard room temperature and atmospheric pressure conditions.

**Figure 5:**
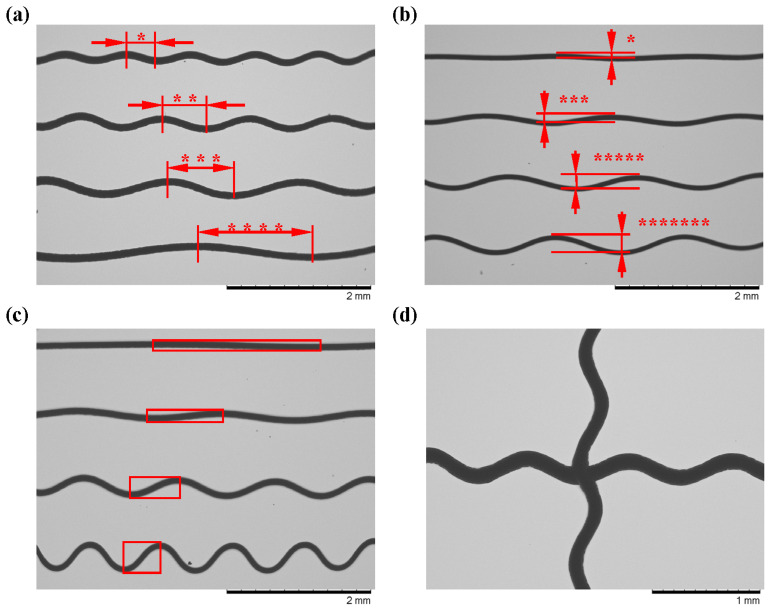
Influence law of printing parameters: (**a**) the printing effect of frequency variation; (**b**) the printing effect of amplitude variation; (the “*” denotes the ratio of the length of the period or peak) (**c**) the printing effect of simultaneous frequency and amplitude variation; (the length and width of the rectangular red frame denote the length of the period and amplitude) (**d**) bidirectional ripple printing.

Prior to investigating the impact of the printing parameters on the deposition pattern, we conducted a preliminary assessment to ascertain the viability of modulating the serpentine structure’s parameters through alterations to the PZT. The results are presented in Figure 5. Figure 5a illustrates the outcomes of modifying the input frequency in accordance with the 1:2:3:4 ratio. Figure 5b depicts the consequences of altering the amplitude in alignment with the 1:3:5:7 ratio, and Figure 5c presents the results for modifying both the frequency and the amplitude simultaneously. It is evident that the trends in the amplitude and period of the fibers exhibit a high degree of uniformity and consistency with the changing trends in the input parameters. Furthermore, Figure 5d illustrates the potential for this technique to be extended to multi-directional printing.

### 3.2. Analysis of the Process Variables

In this study, the default experimental conditions included a nozzle voltage of approximately 2 kV and a platform speed of 5 mm/s.

**Figure 6:**
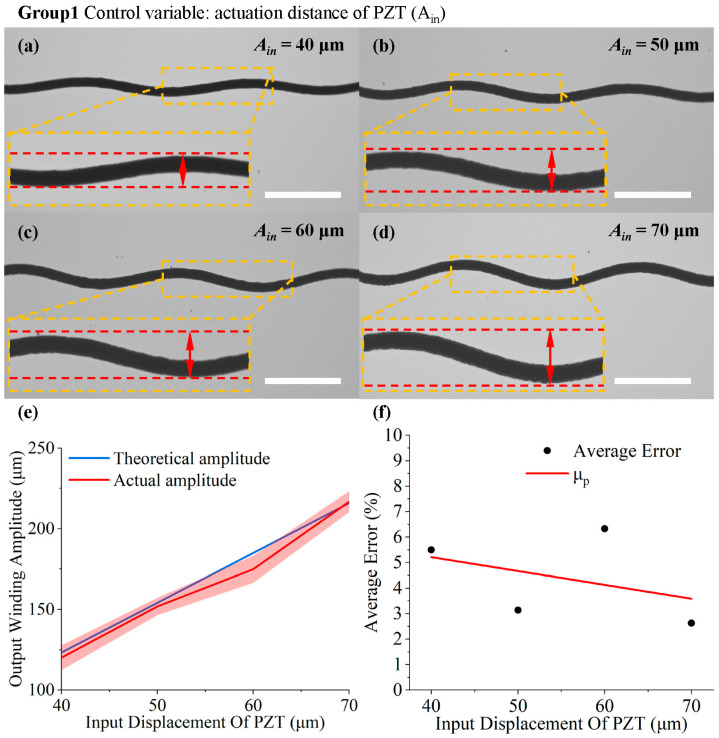
The experimental results for varying amplitude input: (**a**–**d**) deposition waveforms when the output displacement of the PZT rises from 40 μm to 70 μm with a step size of 10 μm (the scale bar is 1 mm); (**e**) the measurement results for the peak height of the fiber varying with the input displacement; (**f**) error analysis and the fitting curve of the average error.

In the first set, Figure 6a–d illustrate the peak height of the serpentine structure with different input amplitudes. As can be seen in Figure 6, the actual peak height obtained through printing is in good agreement with the theoretical value. As the output displacement increases, the theoretical displacement output ranges from 123.32 μm to 215.81 μm, and the actual displacement output increases from 120.10 ± 7.72 μm to 216.77 ± 6.48 μm. Simultaneously, the error bars near the curve of theoretical values indicate the deviation between the actual and theoretical results.

Clearly, in this set, the actual peak value sometimes exceeds or falls short of the theoretical value. Therefore, two scenarios are discussed, in which the actual peak value exceeds or falls short of the theoretical value.

During the direct-writing process, with stable spinning parameters and ambient temperature, the jet deposition is influenced mainly by viscoelastic forces and motion inertia, requiring further clarification: (1) initially, as the solution reaches the molten state, the high surface tension and increasing viscosity make the viscoelastic forces dominant. Meanwhile, the experimental setup is at the critical collection speed for direct writing. When adding PZT micromovement, the synthesis of motion speed in both directions must exceed the critical collection speed of the jet. Consequently, “jet lag” occurs, leading to a “hysteresis effect” in the jet and a smaller actual peak, as illustrated in the supporting material by Liashenko et al. [47]. (2) Additionally, due to the “hysteresis effect”, as the temperature rises, the surface tension decreases, resulting in a coarser jet with increased weight and greater kinematic inertia. During this period, when the PZT elongation reaches its maximum, the jet does not reach the peak trajectory. In this situation, as the PZT contracts, inertia causes the jet to move slightly in the original direction, making the actual peak value greater than the theoretical one. Overall, the error caused in these two cases is not substantial.

As the input displacement increases from 40 μm to 70 μm in steps of 10 μm, the peak errors are 5.50%, 3.14%, 6.33%, and 2.63%, respectively. To compensate for the error, an error coefficient for the peak was determined based on the available experimental results as $\mu_{p}=\left(7.381-0.0542\times A_{in}\right)\times 10^{-2}$.

**Figure 7:**
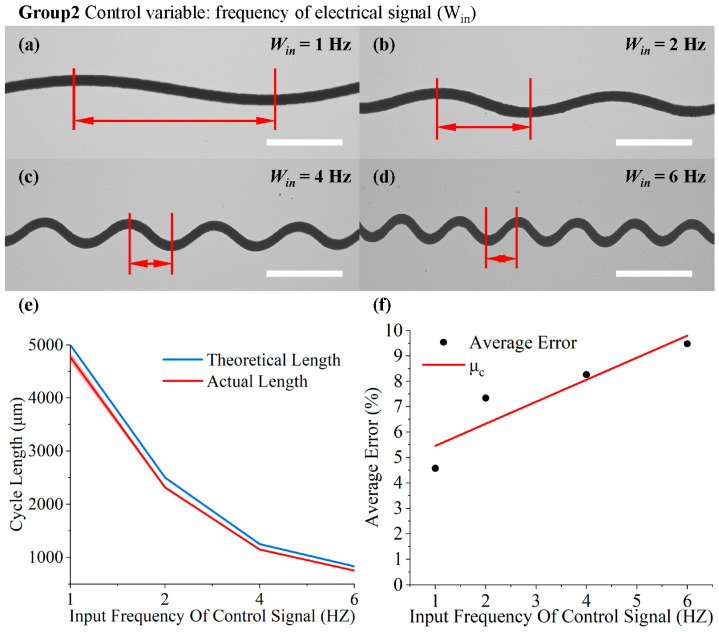
The experimental results for frequency-varying input: (**a**–**d**) deposition waveforms when the input electrical-signal frequency changes from 1 Hz to 6 Hz (the scale bar is 1 mm); (**e**) the measurement results for the bending cycle length of the fiber varying with the input frequency; (**f**) error analysis and the fitting curve of average error.

In the second set, Figure 7a–d demonstrate the variation in the wavelength of the serpentine structure with different input frequencies. As shown in Figure 7e, the theoretical and actual values are numerically similar. With the theoretical output frequency of 1, 2, 4, or 6 Hz and the platform speed of 5 mm/s, the theoretical cycle lengths were 5000, 2500, 1250, and 833 μm, respectively. The actual cycle lengths obtained were about 4771.39 ± 94.16, 2316.485 ± 24.78, 1146.75 ± 7.07, and 754.02 ± 4.95 μm, respectively. There was no significant change in speed in the direction of cycle length, indicating that the speed of PZTs did not change in the direction from elongation to contraction, as observed in the first set of peaks, where hysteresis effects predominated. Simultaneously, as the frequency increased, implying more actuations within the same time frame, the PZT actuation speed rose, amplifying the “hysteresis effect”. This amplification increased the error between the actual results and the theoretical values from 4.57% to 9.48%, demonstrating a parallel trend with the frequency. The correction coefficient for frequency was derived from available experimental results: $\mu_{f}=\left(4.59051-0.86831\times \omega_{in}\right)\times 10^{-2}$.

**Figure 8:**
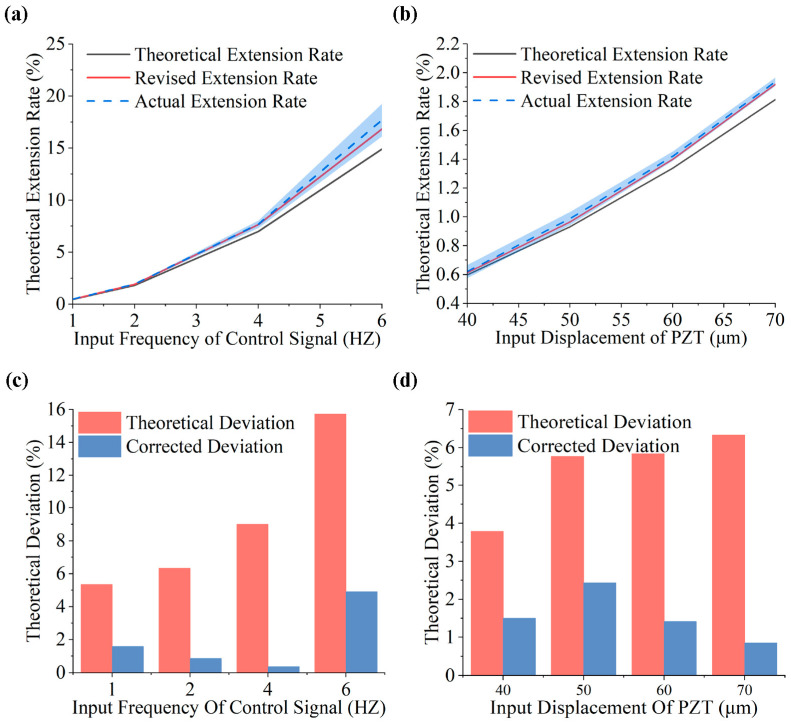
Model calculation and error analysis: (**a**) calculated results for ductility under input-displacement variation; (**b**) measured results for ductility under input-frequency variation; (**c**) errors of the theoretical and corrected results and measured values for varying frequency; (**d**) errors of the theoretical and corrected results and measured values for varying amplitude.

Indeed, the utilization of piezoelectric ceramics as actuators in this study introduces the possibility of thermal effects, environmental factors, and hysteresis characteristics influencing the precision of piezoelectric ceramics’ motion. However, the impact of these discrepancies is relatively minimal, making them challenging to quantify, non-linear, and difficult to circumvent. Consequently, the introduction of the correction coefficient into the calculation model represents a direct and accurate method. By aggregating the potential error into a single correction coefficient, the resulting error coefficient can be utilized to reverse-compensate for the manufacturing errors introduced during the production process. Subsequently, the discrepancy between the theoretical and modified model predictions and the actual lengthening was calculated and analyzed, as shown in Figure 8a,b. The analysis reveals a strong alignment between the actual elongation rates and corrected model predictions. This consistency is observed across varying frequencies and input amplitudes, and the correction falls within the error band for the actual value in all groups, separating it from the theoretical value. To illustrate this, deviations between theoretical, corrected, and actual values were calculated. Figure 8c,d depict the error distribution for the amplitude- and frequency-change groups. Initially, the error distribution for the frequency-change group ranged from 5.34% to 15.73%, while that of the amplitude-change group ranged from 3.79% to 6.33%. After introducing error correction, the model’s computational error was reduced by over 57.86%. The error for the frequency-change group decreased to 0.37–4.90%, while that of the amplitude-change group dropped to 0.85–2.43%. The deviation-corrected results indicate a significant reduction in the discrepancy between the corrected and actual values, with an amplitude decrease ranging from 2.29% to 10.83%. These findings strongly support the accuracy of the theoretical prediction model proposed in this paper and emphasize the necessity of incorporating error correction factors.

**Figure 9:**
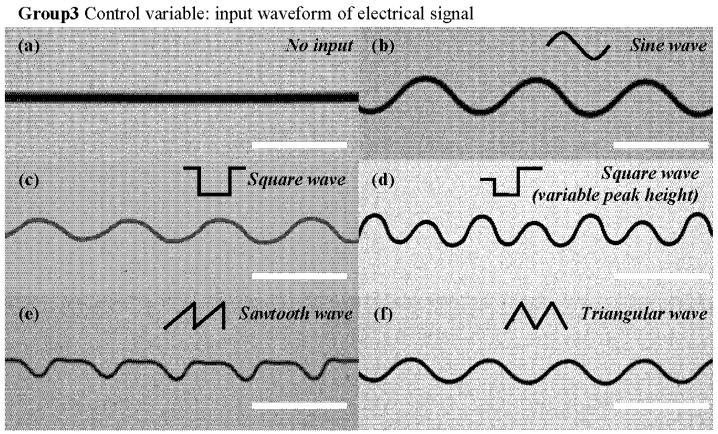
Deposition waveforms with varying input electrical-signal waveforms: (**a**) no input; (**b**) sine wave; (**c**) square wave; (**d**) square wave with variable peak height; (**e**) sawtooth wave; (**f**) triangular wave. (the scale bar is 1 mm).

In the third group, complex serpentine fibers and other waveforms were produced to further demonstrate the process’s expandability. In addition to the common sinusoidal waveform, square, sawtooth, and triangular waveforms were demonstrated. Interestingly, the “hysteresis effect” of the jet causes the printed results from different waveforms to exhibit distinct characteristics. Figure 9c shows the square wave’s printing effect, which resembles a sinusoidal trajectory but with distinct folds between the top and bottom peaks. This can be explained by noting that changing the waveform fundamentally alters the velocity pattern in the PZT. When printing with a square wave, the PZT switches between high and low peaks so rapidly that the jet does not immediately reach the target position, but gradually approaches it during the constant high/low peak segment.

Thus, due to the hysteresis effect, the actual print trajectory of the square wave resembles a sinusoidal trajectory, while retaining the sharp corners caused by the PZT’s rapid movement. Further, Figure 9d demonstrates square wave printing with variable peaks. By reducing the magnitude of the PZT switching amplitude, a more rounded print trajectory is achieved, highlighting the programming flexibility of the method.

Figure 9e presents the sawtooth wave printing effect, blending square- and triangular-wave characteristics, which is evident in the varying peak heights. Finally, Figure 9f displays the triangular waveform results, characterized by smoother peak-height variations, due to the ceramic’s linear actuation being controlled by the triangular waveform.

### 3.3. Mechanical Analysis of Serpentine Structure Fibers

**Figure 10:**
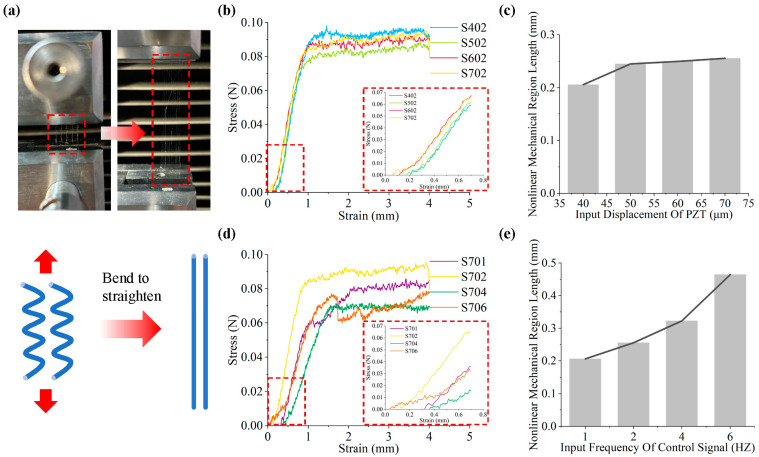
Tensile test results: (**a**) the schematic diagram of fiber stretching; (**b**) fiber stress–strain curve under displacement change; (**c**) length change in the non-linear mechanical region of meandering fiber under displacement change; (**d**) fiber stress–strain curve under frequency change; (**e**) length change in the non-linear mechanical region of meandering fiber under frequency change.

In this section, mechanical stretching experiments were conducted on serpentine structure fibers, and stress–strain curves were plotted for each group based on the experimental results, as shown in Figure 10b,d. A notable feature of the serpentine microstructures is their unique ‘toe region’, highlighted by the red dashed boxes in Figure 10b,d. This represents the mechanical non-linear region that straight fibers cannot achieve during stretching, as demonstrated by Yang et al. [48]. This distinct mechanical non-linear behavior is commonly observed in natural tendons, ligaments, and other mechanically elastic tissues. The mechanism of formation of this mechanical property can be explained as follows [49]: the deformation is dominated by bending due to the initial curvature of the structure. When the serpentine structure is fully elongated into a straight line, the deformation is dominated by axial strain. This transition from bending to straight causes the effective modulus of the serpentine structure to increase with the applied strain, leading to the formation of non-linear regions. Therefore, in theory, the mechanical non-linear region essentially reflects the transition of bent fibers from bending to straightening, as shown in Figure 10a. The degree of curvature, or extensibility, is directly related to the length of the mechanical non-linear region. In other words, the non-linear mechanical properties of a structure can be controlled by adjusting the initial curvature (amplitude, period, or both) of the structure. Figure 10c,e illustrate the variation in the length of the mechanical non-linear region under different parameter changes. These figures show that the length of the mechanical non-linear region increases with the fiber’s extensibility. With the amplitude increased by 10 μm, the length of the mechanical non-linear region exhibited a notable increase, from 0.206 mm to 0.256 mm. This corresponded to an expansion in the toe region, from 2.06% strain to 2.56%. This value was found to be highly comparable to that for the toe region observed in native tendon tissue [50]. The growth trend is subtle yet consistent, aligning with the changes in fiber extensibility induced by the amplitude. Changes in frequency result in a significant increase in plasticity, from 0.48% to 17.70%, which leads to a corresponding increase in the length of the mechanical non-linear region. Thus, this experiment demonstrates the technique’s potential for producing fibers with controllable bending structures and biomimetic mechanical behavior, suitable for tissue engineering applications.

## 4. Conclusions

This study introduced a novel macro–micro-composite NFDW technique utilizing PZT micromotion to programmatically fabricate arrays of serpentine microstructures. The findings can be summarized as follows:

(1) The proposed method markedly enhances the precision of the original platform’s motion, while maintaining its extensive travel range.
(2) The amplitude, frequency, and waveform of the input electrical signals are independently controllable, thereby enabling the precise and accurate fabrication of microstructures.
(3) The impact of diverse process parameters on the geometric configuration of serpentine structures was examined. A theoretical calculation model based on PZT control signals was established, and the introduction of error correction factors resulted in a reduction in computational errors by more than 57.86%. The final mean error of the theoretical model was found to be 1.74%.
(4) The results of mechanical tensile experiments demonstrated that the non-linear mechanical properties of the serpentine structures exhibited a close resemblance to those observed in natural tissues. As the fiber extensibility increased, the length of the non-linear mechanical region of the serpentine structure exhibited a consistent growth pattern.

In conclusion, this work presents a versatile approach that facilitates the precise and reliable deposition of wave-like microstructure arrays in NFDW processes. Building on this foundation, there is considerable potential for the further exploration of this technology. Future work should explore different types of actuators, as each type has distinct motion characteristics that could influence jet deflection and, consequently, printing accuracy. Furthermore, given the significant potential of this method in tissue engineering applications, it would be valuable to investigate the mechanical properties and adjustable ranges of scaffolds made from composite materials. Customizing these materials to meet the mechanical performance requirements of different tissue types is crucial in advancing the practical applications of this technology.

## Figures and Tables

**Table 1:** Performance parameters of PZT and micromotion platforms.

Output Displacement Range of PZT (μm)	Displacement Resolution of PZT (nm)	Displacement Resolution of Macro-Motion Stage (μm)	Displacement Range of Macro-Motion Stage (mm)	Displacement Amplification Ratio (N/μm)
0–95	7	100	100	3.083

**Table 2:** Experimental parameters for NFDW.

Electrode Spacing (mm)	Air Pressure (kPa)	Platform Velocity (mm/s)	Applied Voltage (kV)	Platform Acceleration (mm/s^2^)
2	10~20	4~6	2	200

## Data Availability

The data that support the findings of this study are available from the corresponding authors upon reasonable request.
